# Supplementary material for: Inhibition of TOPORS ubiquitin ligase augments the efficacy of DNA hypomethylating agents through DNMT1 stabilization
Source: Nat Commun. 2024 Aug 28;15:7359. doi: 10.1038/s41467-024-50498-4 (PMC11358161; doi:10.1038/s41467-024-50498-4)
Supplement: Supplementary file 1 — Supplementary Information [file 41467_2024_50498_MOESM1_ESM.pdf]

Supplementary Information to

Kaito et al.;

Inhibition of TOPORS ubiquitin ligase augments the efficacy of DNA hypomethylating agents through DNMT1 stabilization

Extended Data Figure 1-13

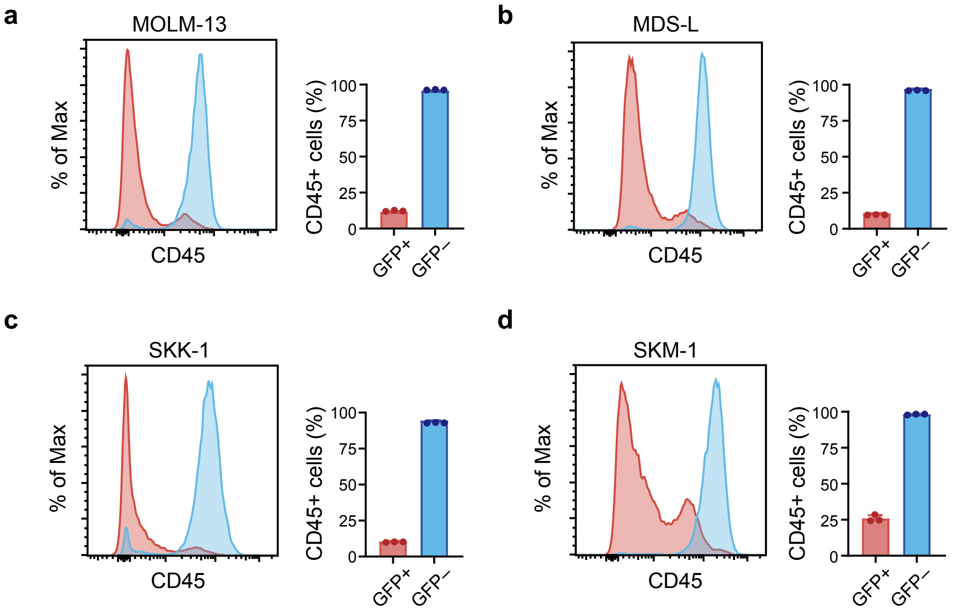

Extended Data Fig. 1 Validation of Cas9 activity in cell lines
The sgRNA against *PTPRC* gene encoding CD45 was transduced using lentivirus and CD45 expression was evaluate 10 days after transduction in (a) MOLM-13, (b) MDS-L, (c) SKK-1, and (d) SKM-1 cells. (n=3, technical replicates, Data derived from a single experiment). Representative flow cytometric profiles and percentage of CD45+ cells are depicted (left and right panels, respectively). P value was calculatd by unpaired two-tailed Student' s *t*-test. Source data are provided as a Source Data file.

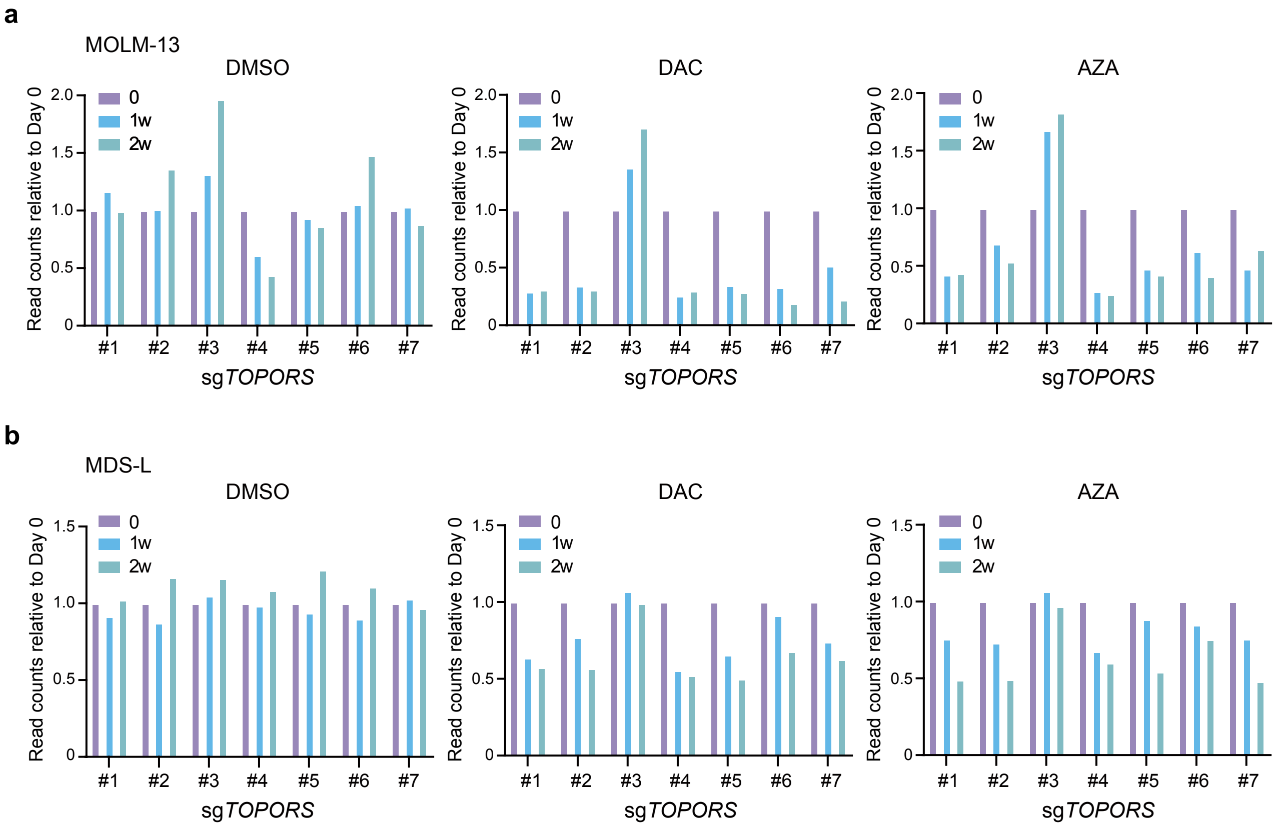

Extended Data Fig. 2. Profiles of sgRNAs against *TOPORS* in the screening
Changes in read counts of sgRNA against *TOPORS* relative to those at day 0 in the screening of MOLM-13 (a) and MDS-L (b) cells are depicted.

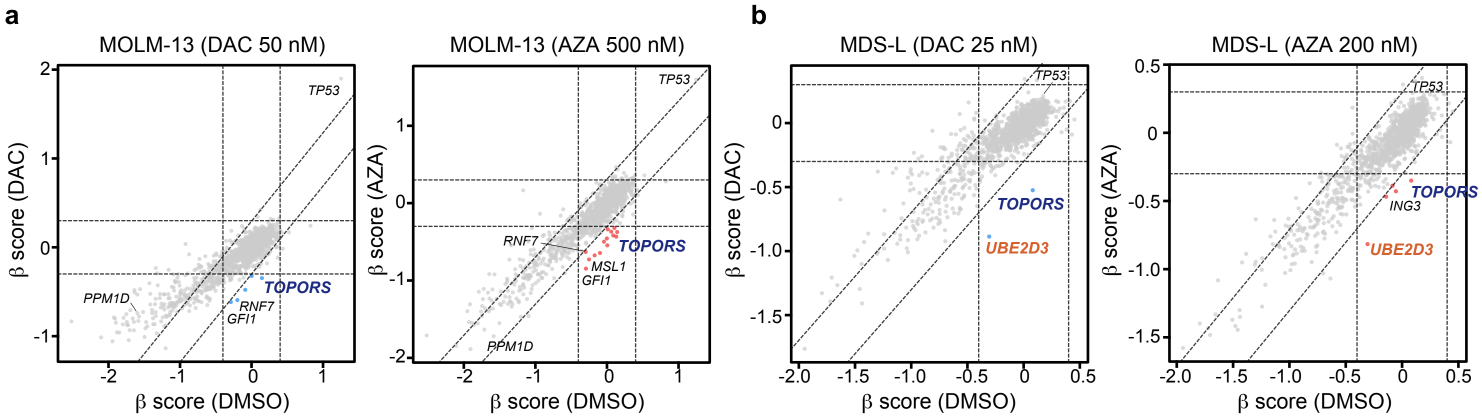

Extended Data Fig. 3. CRISPR-Cas9 screening in the presence of high concentrations of HMAs.
(a, b) CRISPR-Cas9 screening data at high concentrations of HMAs: MOLM-13 cells with DAC at 50 nM and AZA at 500 nM (a) and MDS-L cells with DAC at 25 nM and AZA at 200 nM (b). Scatter plots showing the β scores of each gene, which indicate the changes of sgRNA contents for each gene, in the presence and absence of HMAs. The candidate genes whose sgRNAs decreased during culture specifically in the presence of HMAs are indicated in blue (DAC) and red (AZA) dots.

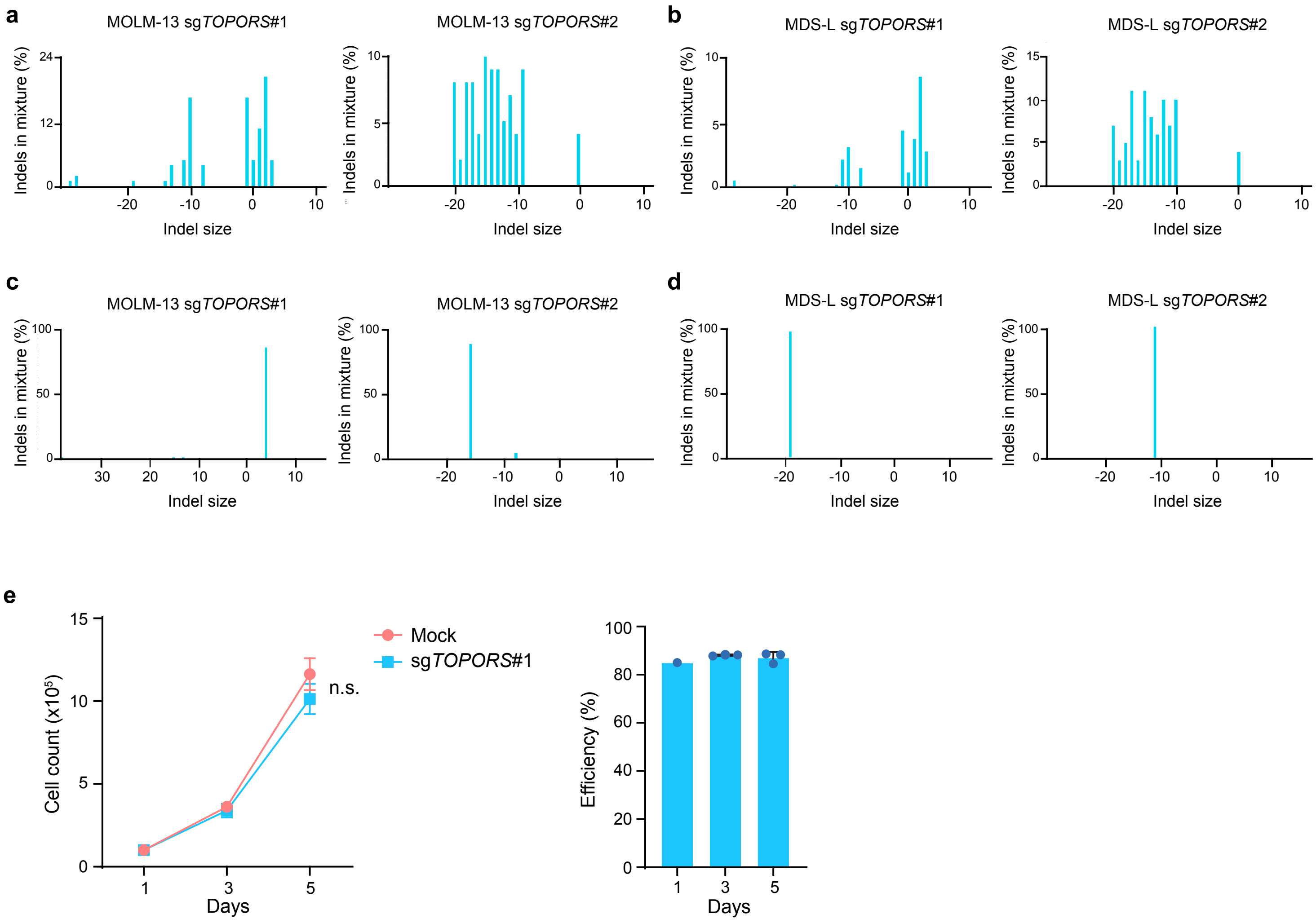

Extended Data Fig. 4. Gene editing profiles of *TOPORS* and impact of *TOPORS* knockout on human HSPCs
(a-d) Gene editing profiles of the *TOPORS* gene obtained by Inference of CRISPR Edits (ICE) analysis using a software tool (Synthego) in bulk *TOPORS*-KO MOLM-13 (a) and MDS-L (b) cells and in *TOPORS*-KO MOLM-13 (c) and MDS-L (d) single clones.
(e) Growth of human CD34-positive HSPCs upon *TOPORS* knockout by gene editing (left panel). Gene editing efficiency is shown in the right panel (n=3, biological replicates). n.s., not significant by the Student’ s *t*-test.

a

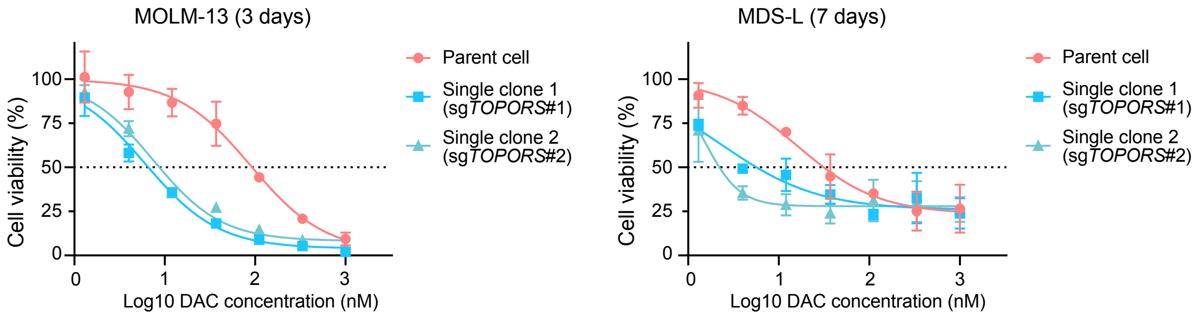

Absolute IC50
WT: 92.44 nM, KO1: 6.581 nM, KO2: 8.716 nM

Absolute IC50
WT: 31.37 nM, KO1: 5.690 nM, KO2: 2.214 nM

b

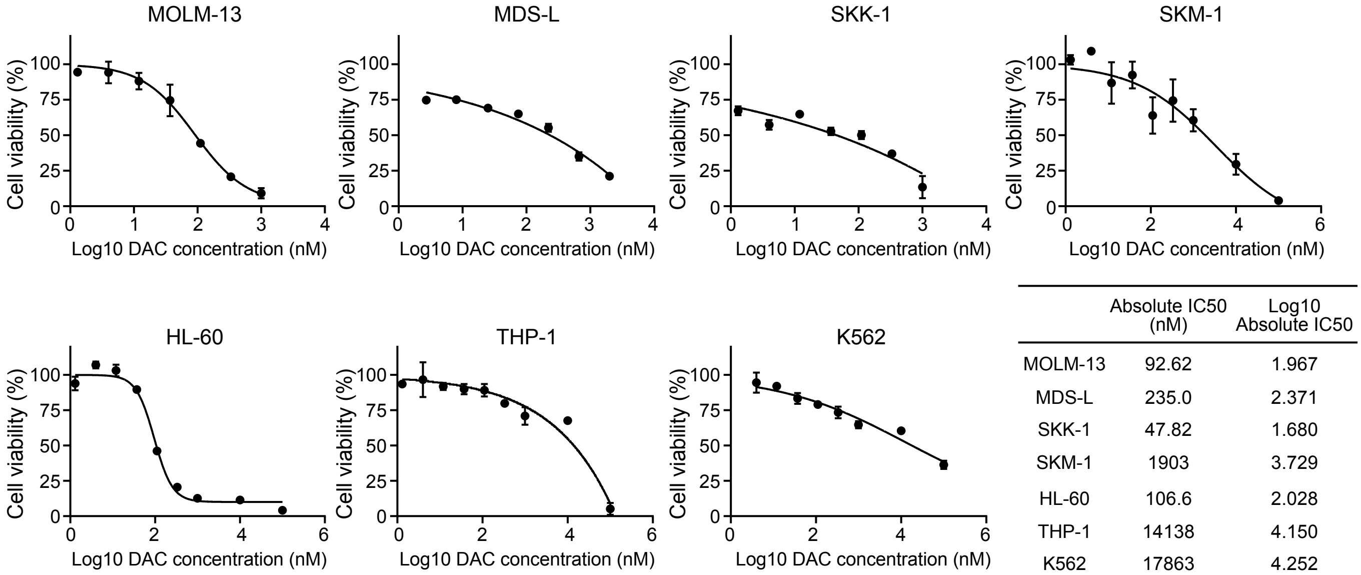

	Absolute IC50 (nM)	Log10 Absolute IC50
MOLM-13	92.62	1.967
MDS-L	235.0	2.371
SKK-1	47.82	1.680
SKM-1	1903	3.729
HL-60	106.6	2.028
THP-1	14138	4.150
K562	17863	4.252

c

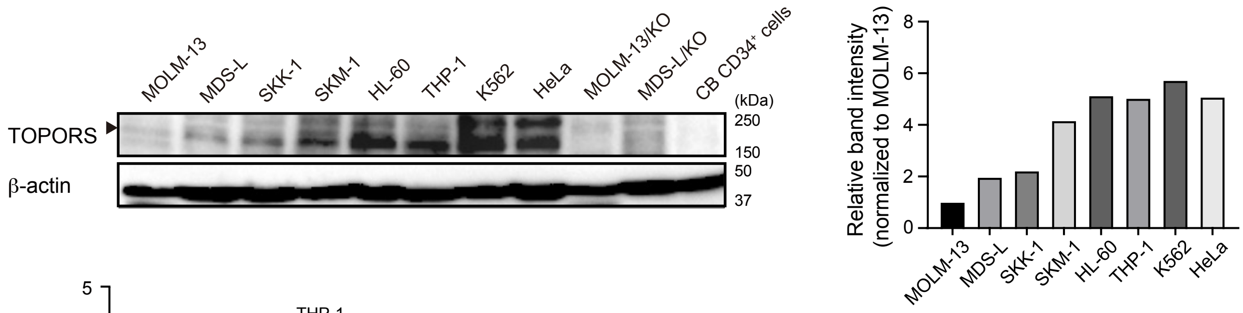

d

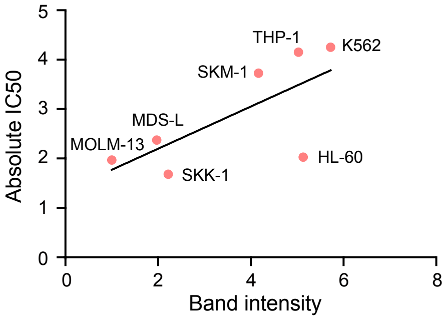

Extended Data Fig. 5. Relationship between DAC resistance and TOPORS protein levels

(a) Dose-response curves of DAC in WT and *TOPORS*-KO MOLM-13 and MDS-L cells. MTS assays were performed 3 and 7 days after DAC exposure to MOLM-13 and MDS-L, respectively to evaluate cell viability (n=3, technical replicates, the experiments were repeated twice independently). Dose-response curve to decitabine (DAC) and absolute IC50 values are indicated.

(b) Dose-response curve to DAC of each cell line (n=3, technical replicates, the experiments were repeated twice independently). Absolute IC50 and Log10 (absolute IC50) values are summarized in a table.

(c) Protein levels of TOPORS (indicated by arrowheads) detected by western blotting in each cell line (arrowheads). MOLM-13/*TOPORS* KO and MDS-L/*TOPORS* KO cells were served as negative controls. Normalized protein levels relative to that in MOLM-13 is depicted in right panel. Protein levels were normalized by that of β-actin and then compared to that of MOLM-13. The samples derive from the same experiment but different gels were processed in parallel.

(d) Correlation of TOPORS protein levels and absolute IC50 of DAC in each cell line.

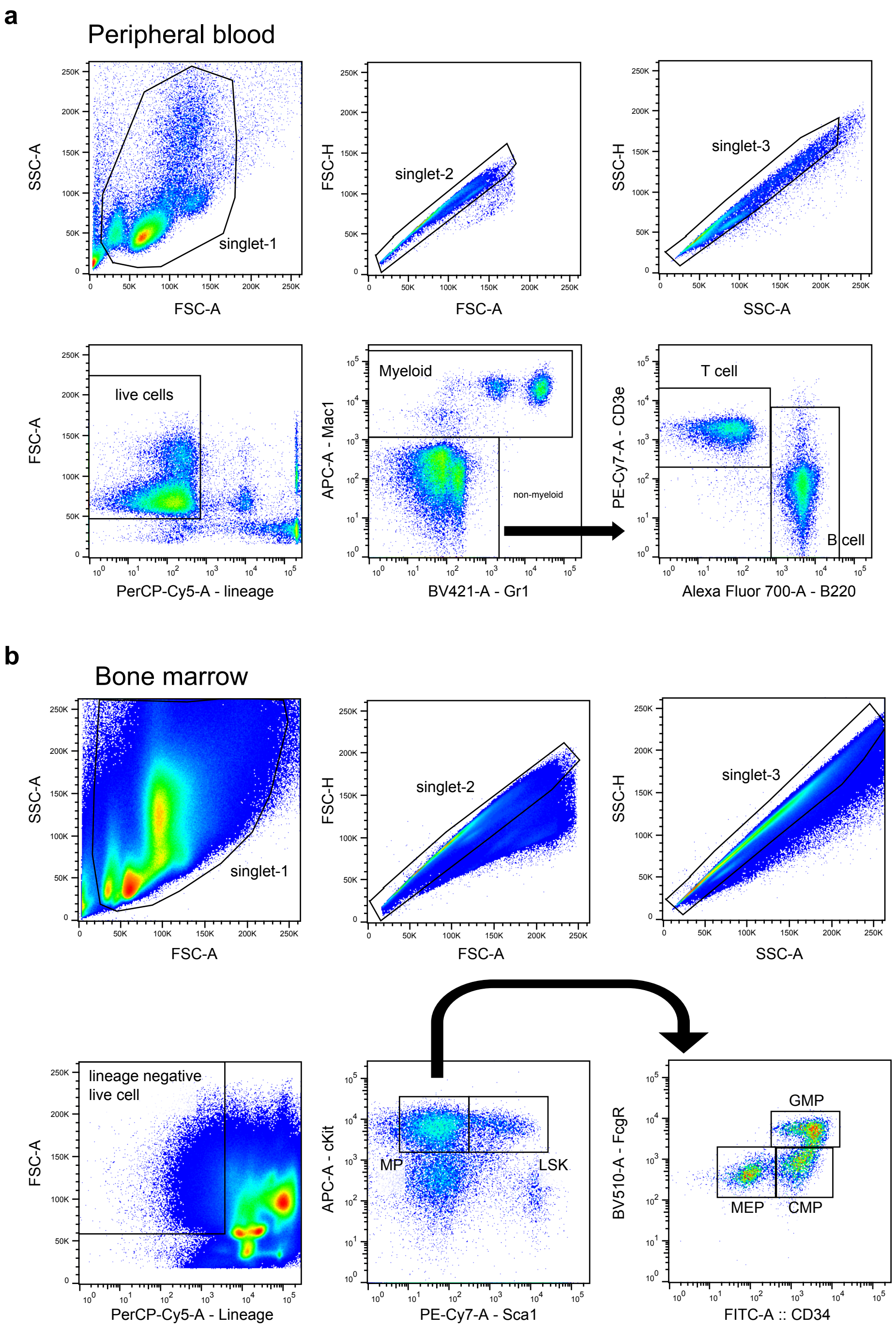

Extended Data Fig. 6. Flow cytometric profiles of *Topors* KO PB and BM hematopoietic cells
(a) Flow cytometric profiles of myeloid cells and T and B cells in the PB of WT and *Topors* KO mice.
(b) Flow cytometric profiles of LSK cells, MPs, CMPs, GMPs, and MEPs in the BM of WT and *Topors* KO mice.

a

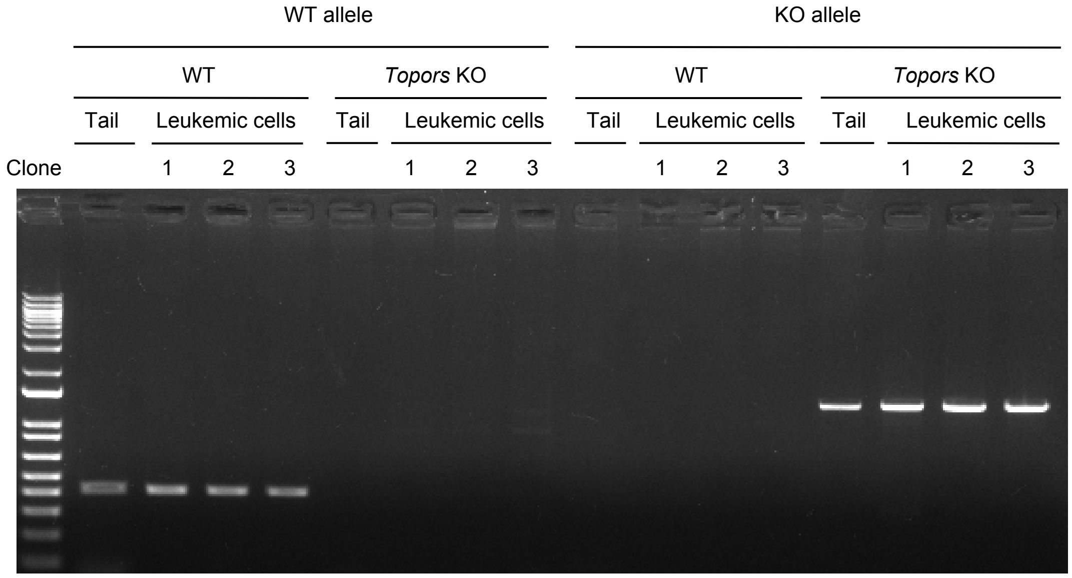

b

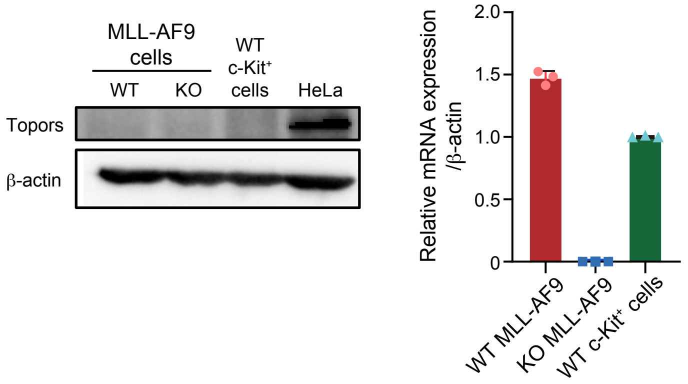

Extended Data Fig. 7. Expression of *Topors* in *MLL-AF9* leukemic cells

(a) Genotypes of *MLL-AF9* leukemic cells derived from WT and *Topors* KO mice confirmed by PCR. Tail DNA was used as controls.

(b) Topors protein and *Topors* mRNA levels in *MLL-AF9* leukemic cells and WT c-Kit$^+$ HSPCs assed by western blotting (left) and RT-qPCR (n=3) (right). HeLa cell lysates were used as a control in western blotting. The samples derive from the same experiment but different gels were processed in parallel. mRNA expression of *Topors* was normalized by β-actin mRNA (n=3, technical replicates, the experiments were repeated twice independently). ***p < 0.001 by unpaired two-tailed Student' s *t*-test. Data are presented as mean ± SD. Source data are provided as a Source Data file.

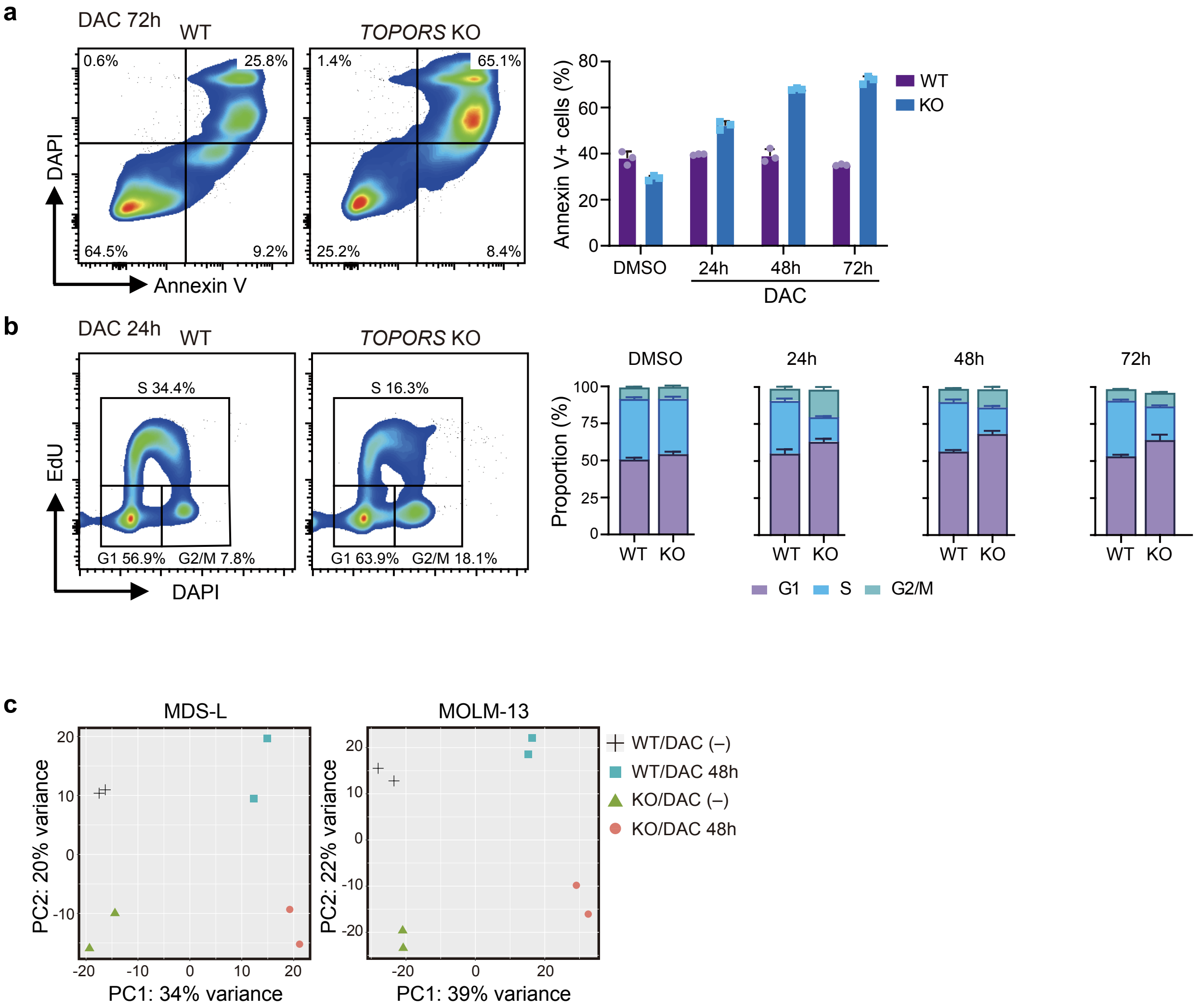

Extended Data Fig. 8. Apoptosis and cell cycle assays of MOLM-13 cells
(a) Frequency of apoptotic cell death in WT and *TOPORS*-KO MOLM-13 cells after exposure to 20 nM of DAC. Representative flow cytometric profiles of cells at 72 hours of DAC exposure (left panel). Percentage of Annexin V-positive cells (n=3, technical replicates, the experiments were repeated twice independently) (right panel).
(b) Cell cycle status of WT and *TOPORS*-KO MOLM-13 cells after exposure
to 12.5 nM of DAC evaluated by EdU and DAPI. Representative flow cytometric profiles of cells at 72 hours of DAC exposure (left panel). Proportion of each cell cycle at the indicated time points (n=3, technical replicates, the experiments were repeated twice independently) (right panel).
*p < 0.05; **p < 0.01; ***p < 0.001; n.s., not significant by unpaired two-tailed Student' s *t*-test.
Data are presented as mean ± SD. Source data are provided as a Source Data file.

(c) RNA sequence analysis data of MDS-L and MOLM-13 cells. PCA plots of RNA sequencing data of WT and *TOPORS*-KO cells in the presence and absence of DAC.

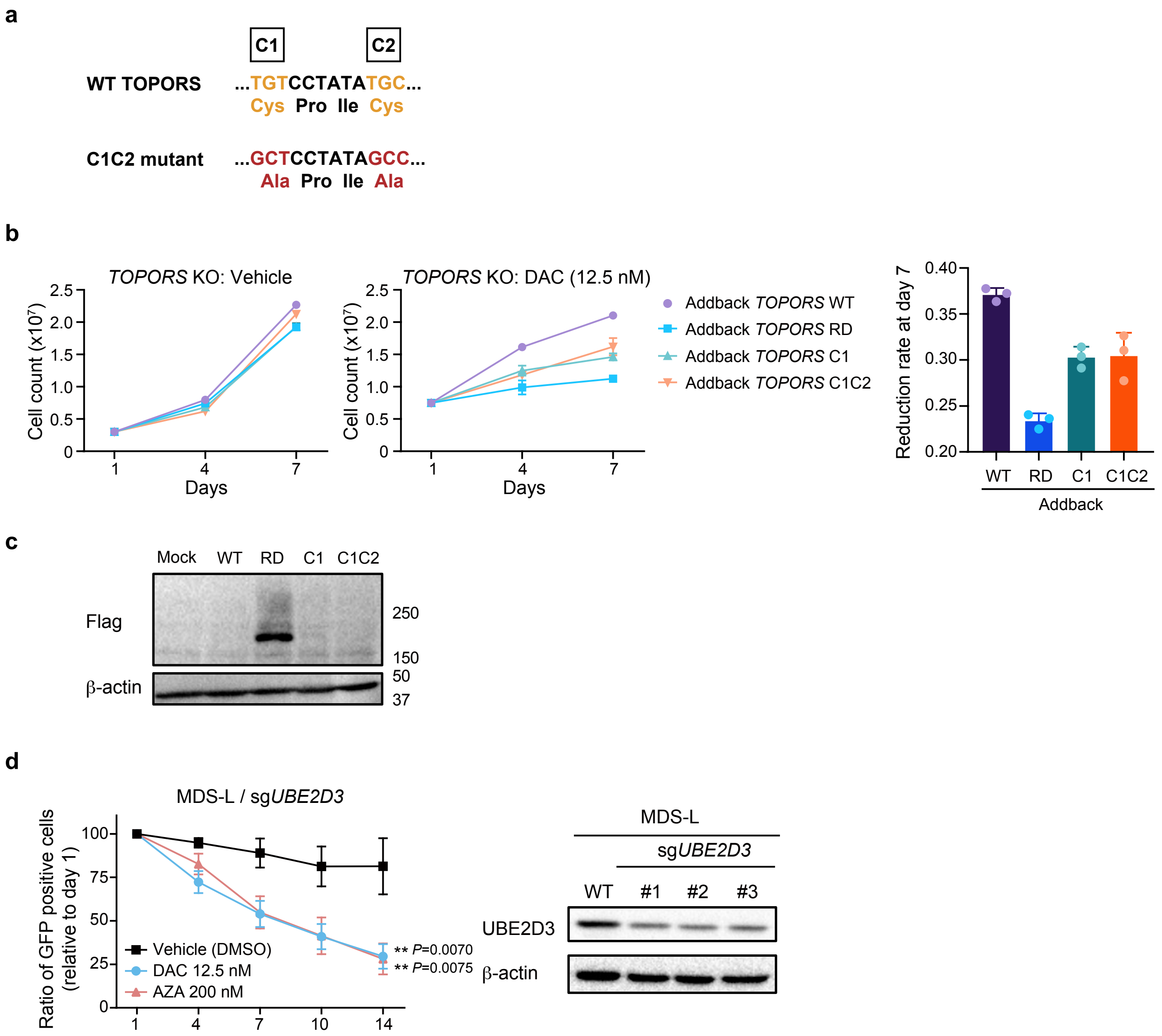

Extended Data Fig. 9. Effects of TOPORS C1 and C1C2 mutants and *UBE2D3* knockout
(a) Design of C1 and C1C2 mutants in the RING finger domain.
(b) Effects of C1 and C1C2 mutant add-back on TOPORS-KO MDS-L growth in the presence and absence of 12.5 nM DAC. Growth curve (left) and reduction rate of cell numbers at day 7 of culture relative to those of WT MDS-L cells (right) (n=3, technical replicates, the experiments were repeated twice independently). *p < 0.05; **p < 0.01; ***p < 0.001; n.s., not significant by unpaired two-tailed Student' s *t*-test.
(c) Protein levels of TOPORS and TOPORS mutants detected by western blotting. β-actin was served as a loading control. The samples derive from the same experiment but different gels were processed in parallel.
(d) Effect of *UBE2D3* KO on the growth of MDS-L cells in the presence and absence of DAC or AZA. Three different sgRNAs against *UBE2D3* were tested in triplicate and combined data are shown. Western blot data of UBE2D3 is depicted in the right panel. **p < 0.01 by unpaired two-tailed Student' s *t*-test. Data are presentted as mean ± SD.
Source data are provided as a Source Data file.

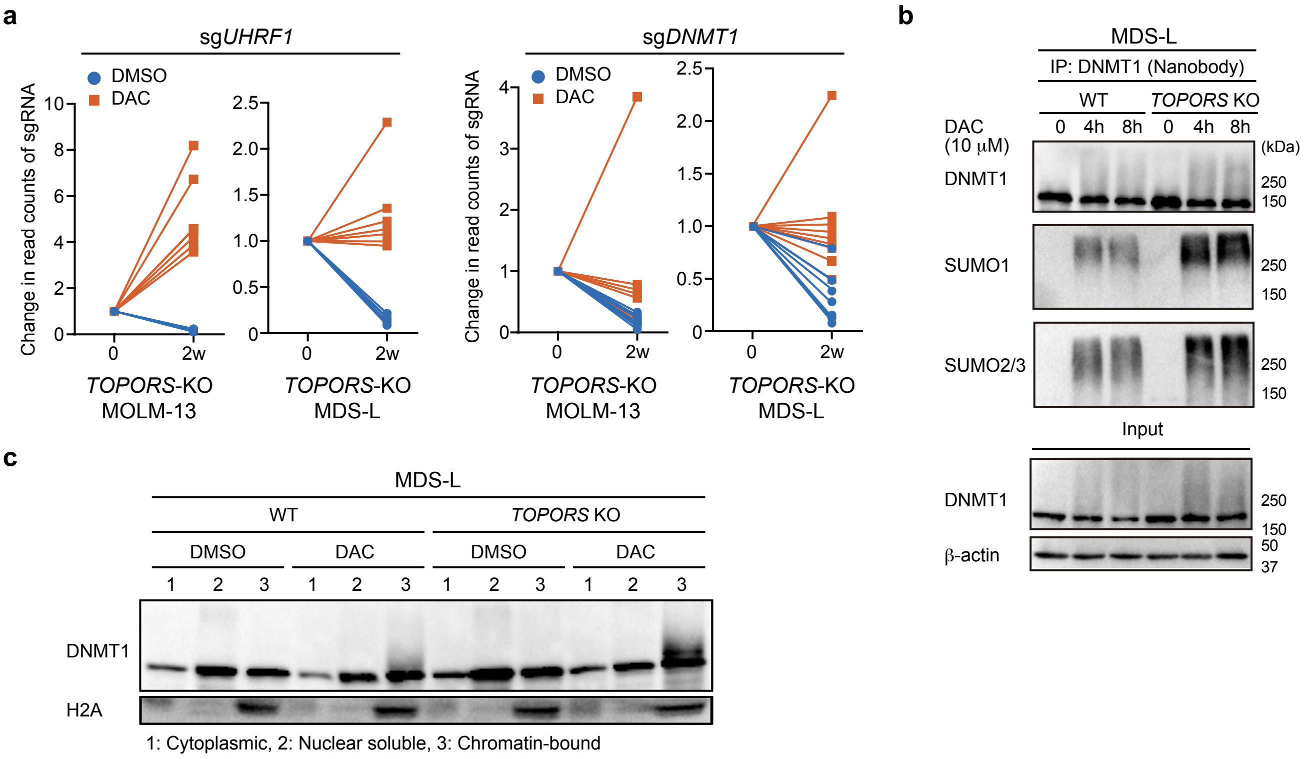

Extended Data Fig. 10. sgRNA profiles against *DNMT1* (2nd screening) and DNMT1 modifications

(a) Changes in read counts of sgRNA against *UHRF1* and *DNMT1* before and after the second screening of *TOPORS-KO* MOLM-13 (left) and MDS-L (right) cells.

(b) SUMOylation and ubiquitination levels of DNMT1 in WT and *TOPORS*-KO MDS-L cells treated with 10 nM DAC for indicated hours. Endogenous DNMT1 immunoprecipitated with anti-DNMT1 nanobody was subjected to western blotting. β-actin was served as a loading control of the inputs. The samples derive from the same experiment but different gels for DNMT1, β-actin, another for DNMT1 (ip), another for SUMO1, and another for SUMO2/3 were processed in parallel.

(c) Western blot analysis of DNMT1 in subfractions of MDS-L cells after exposure to high-dose DAC (10 μM) for 8 hours. H2A was served as a loading control of the inputs. The samples derive from the same experiment but different gels were processed in parallel.

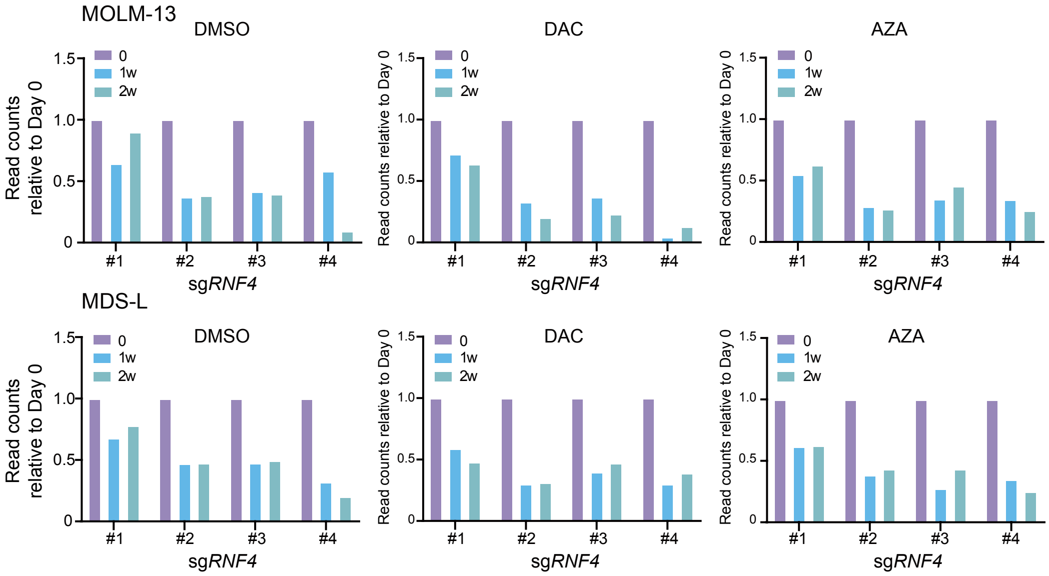

Extended Data Fig. 11. Profiles of sgRNA against *RNF4* in the first screening
Changes in read counts of sgRNA against *RNF4* relative to those at day 0 in the the first screening of MOLM-13 and MDS-L cells.

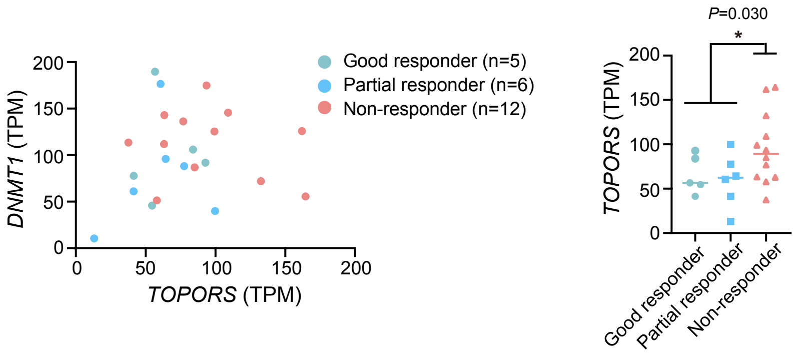

Extended Data Fig. 12. Expression of *TOPORS* in primary MDS/AML

The correlation between the expression levels of *TOPORS* and *DNMT1* in BM mononuclear cells (left) and the response to AZA treatment (right) in 23 patients with MDS or AML.

*p < 0.05 by unpaired two-tailed Student’ s *t*-test. Source data are provided as a Source Data file.

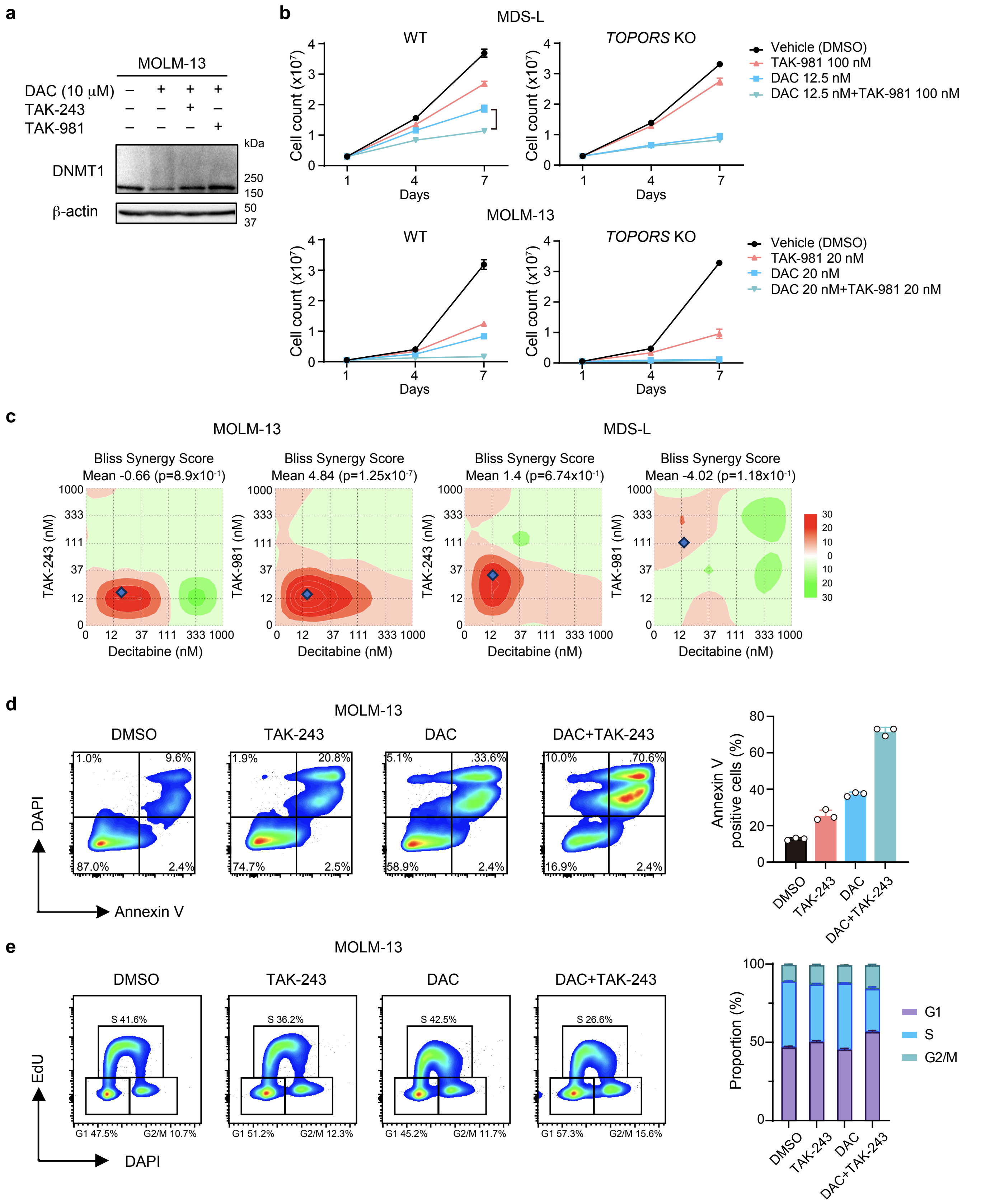

Extended Data Fig. 13. Pharmacological intervention induces mitotic defects via DNMT1 stabilization
(a) Changes in DNMT1 protein levels after exposure to DAC in MOLM-13 cells. The cells were exposed to a high dose of DAC (10 μM) for 8 hours in the presence and absence of TAK-243 (1 μM) or TAK-981 (3 μM).
(b) Growth of MDS-L and MOLM-13 clones in the presence and absence of DAC and/or TAK-981 (n=3, technical replicates, the experiments were repeated twice independently).
(c) Synergy effects between DAC and TAK-243 or TAK-981 assessed in MOLM-13 and MDS-L at various combinations of concentrations. Synergy was calculated by SynergyFinder 3.0, and BLISS was used to denote scores. Representative combinations of concentrations at which the synergistic effect was particularly strong are shown by dots.
(d) Frequency of apoptotic cell death in MOLM-13 cells after exposure to 12.5 nM of DAC. Representative flow cytometric profiles of cells at 72 hours of DAC exposure (left panel). Percentage of Annexin V-positive cells (n=3, technical replicates, the experiments were repeated twice independently) (right panel).
(e) Cell cycle status of MOLM-13 cells 48 hours after exposure to DAC (20 nM) and/or TAK-243 (20 nM). Representative flow cytometric profiles of cells (left panel). Proportion of each cell cycle was evaluated by EdU incorporation and DAPI staining 48 hours after treatment (n=3, technical replicates, the experiments were repeated twice independently) (right panel).
*$p < 0.05$; **$p < 0.01$; ***$p < 0.001$; n.s., not significant by the Student’ s *t*-test. Data are presentted as mean ± SD.
Source data are provided as a Source Data file.
