# Supplementary material for: Inhibition of TOPORS ubiquitin ligase augments the efficacy of DNA hypomethylating agents through DNMT1 stabilization
Source: Nat Commun. 2024 Aug 28;15:7359. doi: 10.1038/s41467-024-50498-4 (PMC11358161; doi:10.1038/s41467-024-50498-4)
Supplement: Supplementary file 3 — Description of Additional Supplementary Information [file 41467_2024_50498_MOESM3_ESM.docx]

**Description of Additional Supplementary Files**

File Name: Supplementary Data 1

Description: Characteristics of cell lines

File Name: Supplementary Data 2

Description: 1st screening data (candidate gene list)

File Name: Supplementary Data 3

Description: DEG list

File Name: Supplementary Data 4

Description: DEG list

File Name: Supplementary Data 5

Description: GO terms enriched in ubiquitinated peptides identified in WT MDS-L cells compared to those in TOPORS KO MDS-L cells

File Name: Supplementary Data 6

Description: DNMT1-derived peptides detected in mass spectrometric analysis of WT and TOPORS KO MDS-L cells in the presence of DAC

File Name: Supplementary Data 7

Description: Patients' profiles

File Name: Supplementary Data 8

Description: Primer sequences for amplification of sgRNA portions and sequencing by next generation sequencing
