# Supplementary material for: Inhibition of TOPORS ubiquitin ligase augments the efficacy of DNA hypomethylating agents through DNMT1 stabilization
Source: Nat Commun. 2024 Aug 28;15:7359. doi: 10.1038/s41467-024-50498-4 (PMC11358161; doi:10.1038/s41467-024-50498-4)
Supplement: Supplementary file 12 — Reporting Summary [file 41467_2024_50498_MOESM12_ESM.pdf]

nature portfolio

Corresponding author(s): Atsushi Iwama

Last updated by author(s): 2024/06/03

Reporting Summary

Nature Portfolio wishes to improve the reproducibility of the work that we publish. This form provides structure for consistency and transparency in reporting. For further information on Nature Portfolio policies, see our Editorial Policies and the Editorial Policy Checklist.

Statistics

For all statistical analyses, confirm that the following items are present in the figure legend, table legend, main text, or Methods section.

n/a	Confirmed	
☐	☒	The exact sample size (*n*) for each experimental group/condition, given as a discrete number and unit of measurement
☐	☒	A statement on whether measurements were taken from distinct samples or whether the same sample was measured repeatedly
☐	☒	The statistical test(s) used AND whether they are one- or two-sided<br>*Only common tests should be described solely by name; describe more complex techniques in the Methods section.*
☒	☐	A description of all covariates tested
☐	☒	A description of any assumptions or corrections, such as tests of normality and adjustment for multiple comparisons
☐	☒	A full description of the statistical parameters including central tendency (e.g. means) or other basic estimates (e.g. regression coefficient) AND variation (e.g. standard deviation) or associated estimates of uncertainty (e.g. confidence intervals)
☐	☒	For null hypothesis testing, the test statistic (e.g. *F*, *t*, *r*) with confidence intervals, effect sizes, degrees of freedom and *P* value noted<br>*Give P values as exact values whenever suitable.*
☒	☐	For Bayesian analysis, information on the choice of priors and Markov chain Monte Carlo settings
☒	☐	For hierarchical and complex designs, identification of the appropriate level for tests and full reporting of outcomes
☒	☐	Estimates of effect sizes (e.g. Cohen's *d*, Pearson's *r*), indicating how they were calculated

Our web collection on statistics for biologists contains articles on many of the points above.

Software and code

Policy information about availability of computer code

Data collection	Detailed information is described in the method section. Briefly, Hiseq 2500 (Illumina) was used for CRISPR/Cas9 screening and RNA sequence. FACS Celesta (BD) and FACS Aria III (BD) were used for cell analysis and sorting. Image lab (BioLad) was used for western blot quantification. Orbitrap Eclipse Tribrid mass spectrometer with a FAIMS Pro interface (Thermo Fisher Scientific) was used for mass spectrometry.
Data analysis	Softwares used in this study were described in the method section. Briefly, Prism GraphPad 9 (GraphPad) was used to analyze the data and create the figure. Flow jo 10.1 (FlowJo, LCC) was used to analyze flow cytometry plots. RNA sequence data was analyzed with iDEP95 (Ge et al., 2018). Proteome Discoverer 2.5 (Thermo Fisher Scientific) was used to analyze the mass spectrometry data. Gene set enrichment analysis was performed with GSEA 4.3.0 (Broad Institute). CRISPR/Cas9 screening results were analyzed using MAGeCK (Liu et al., 2014). SUMO-interaction motifs was predicted with GPS-SUMO (Zhao et al., 2014).

For manuscripts utilizing custom algorithms or software that are central to the research but not yet described in published literature, software must be made available to editors and reviewers. We strongly encourage code deposition in a community repository (e.g. GitHub). See the Nature Portfolio guidelines for submitting code & software for further information.

Data

Policy information about availability of data

All manuscripts must include a data availability statement. This statement should provide the following information, where applicable:

- Accession codes, unique identifiers, or web links for publicly available datasets
- A description of any restrictions on data availability
- For clinical datasets or third party data, please ensure that the statement adheres to our policy

The RNA sequence data were deposited to the DNA Data Bank of Japan (accession number: DRA015263). The mass spectrometry proteomics data were deposited to the ProteomeXchange Consortium via the jPOST repository with the dataset identifier PXD044347 and 048275.

Research involving human participants, their data, or biological material

Policy information about studies with human participants or human data. See also policy information about sex, gender (identity/presentation), and sexual orientation and race, ethnicity and racism.

Reporting on sex and gender	Sex and gender of participants was determined based on self-report but was not considered in this study.
Reporting on race, ethnicity, or other socially relevant groupings	Only Japanese samples were used.
Population characteristics	MDS and AML samples with and without treatment with Azacitidine
Recruitment	MDS and AML patients were recruited without any bias. Informed consent was obtained by participants.
Ethics oversight	The institutional ethical committee at Kyoto University (Approval #G-608) and Tokyo Metropolitan Cancer and Infectious Diseases Center, Komagome Hospital (Approval #2203). informed consent was obtained by participants.

Note that full information on the approval of the study protocol must also be provided in the manuscript.

Field-specific reporting

Please select the one below that is the best fit for your research. If you are not sure, read the appropriate sections before making your selection.

☒ Life sciences ☐ Behavioural & social sciences ☐ Ecological, evolutionary & environmental sciences

For a reference copy of the document with all sections, see nature.com/documents/nr-reporting-summary-flat.pdf

Life sciences study design

All studies must disclose on these points even when the disclosure is negative.

Sample size	Sample size was determined based on previous paper (Joung et al., Nat protocol 2017).
Data exclusions	All sequence data were included in this analysis.
Replication	The CRISPR screening data were reproducible in screening at different drug concentrations using two different cell lines (supplementary figure 3. The results was validated and also in individually designed sgRNAs (figure 2).
Randomization	In xenograft experiments, recipient mice engrafted by human leukemic cells were randomized based on the leukemic cell burden in the peripheral blood.
Blinding	The researcher was not blinded. The subjectivity of the author would not have affected the analysis in this study.

Reporting for specific materials, systems and methods

We require information from authors about some types of materials, experimental systems and methods used in many studies. Here, indicate whether each material, system or method listed is relevant to your study. If you are not sure if a list item applies to your research, read the appropriate section before selecting a response.

Materials & experimental systems

n/a	Involved in the study	
☐	☒	Antibodies
☐	☒	Eukaryotic cell lines
☒	☐	Palaeontology and archaeology
☐	☒	Animals and other organisms
☐	☒	Clinical data
☒	☐	Dual use research of concern
☒	☐	Plants

Methods

n/a	Involved in the study	
☒	☐	ChIP-seq
☐	☒	Flow cytometry
☒	☐	MRI-based neuroimaging

Antibodies

Antibodies used

PE Anti-human CD45 (clone HI30) BioLegend 304039; RRID: AB_2562057
e450 Anti-mouse Ly-6G/Ly-6C (clone RB6-8C5) Invitrogen 48-5931-82; RRID: AB_1548788
APC Anti-mouse CD11b (clone M1/70) BioLegend 101212; RRID: AB_ 312795
PE/Cy5 Anti-mouse Ter119 (clone TER-119) Invitrogen 15-5921-82; RRID: AB_468810
AF700 Anti-mouse B220 (clone RA3-6B2) BioLegend 103232; RRID: AB_493717
PE/Cy7 Anti-mouse CD3e (clone 145-2C11) BioLegend 100320; RRID: AB_312685
FITC Anti-mouse CD45.2 (clone 104) BioLegend 109806; RRID: AB_313443
APC/Cy7 Anti-mouse CD45.1 (clone A20) BioLegend 110716; RRID: AB_313505
PE/Cy5 Anti-mouse CD3e (clone 145-2C11) Invitrogen 15-0031-83; RRID: AB_468690
PE/Cy5 Anti-mouse CD4 (clone GK1.5) BioLegend 100409; RRID: AB_312694
PE/Cy5 Anti-mouse CD5 (clone 53-7.3) BioLegend 100610; RRID: AB_312739
PE/Cy5 Anti-mouse CD8a (clone 53-6.7) BioLegend 100710; RRID: AB_312749
PE/Cy5 Anti-mouse B220 (clone RA3-6B2) BioLegend 103209; RRID: AB_312994
PE/Cy5 Anti-mouse CD11b (clone M1-70) BioLegend 101210; RRID: AB_312793
PE/Cy5 Anti-mouse Ly-6G/Ly-6C (clone RB6-8C5) BioLegend 108410; RRID: AB_313375
APC Anti-mouse cKit (CD117) (clone 2B8) BioLegend 105812; RRID: AB_313221
PE/Cy7 Anti-mouse Ly-6A/E (Sca-1) (clone D7) BioLegend 108114; RRID: AB_493596
FITC Anti-mouse CD34 (clone RAM 34) Invitrogen 11-0341-85; RRID: AB_465021
e450 Anti-mouse CD34 (clone RAM34) Invitrogen 48-0341-82; RRID: AB_2043837
BV510 Anti-mouse CD16/32 (FcgRIII/II) (clone 93) BioLegend 101333; RRID: AB_2563692
APC Annexin V BD Biosciences 550475; RRID: AB_2868885
Anti-B-actin Cell Signaling Technology 4967; RRID: AB_330288
Anti-TOPORS Bethyl Laboratories A302-179A; RRID: AB_1659846
Anti-DNMT1 (D63A6) Cell Signaling Technology 5032; RRID: AB_10548197
Anti-UHRF1 (clone 28) BD Biosciences 612264; RRID: AB_399581
Anti-SUMO1 Cell Signaling Technology 4930; RRID: AB_10698887
Anti-SUMO2/3 Abcam ab3742; RRID: AB_304041
Anti-Ubiquitin (P4D1) Santa Cruz Biotechnology sc-8017; RRID: AB_628423
Anti-Rabbit IgG, HRP-Linked Cytiva NA934
Anti-Mouse IgG, HRP-Linked Cytiva NA933

Validation

Combination of these antibodies were already utilized by many published articles.
Nat Commun 12(1):3568, 2021.
J Exp Med 218(3):e20192283, 2021.
Leukemia 35:1156-1165, 2021.
Blood 128(5):638-649, 2016.
Exp Hematol 44:282-296, 2016.
Blood 126:1172-1183, 2015

All antibodies are validated by company.

Eukaryotic cell lines

Policy information about cell lines and Sex and Gender in Research

Cell line source(s)

MOLM-13 DSMZ ACC 544
MDS-L (Gift from Kaoru Tohyama) PMID: 29955132
SKK-1 (Gift from Hiroshi Matsuoka) PMID: 15996938
SKM-1 DSMZ ACC 547
Human CB-derived MLL-AF9 leukemic cells (Gift from Susumu Goyama) PMID: 36453131

Authentication

None of these cells used in this study were authenticated.

Mycoplasma contamination	These cell lines were not tested for Mycoplasma contamination.
Commonly misidentified lines (See ICLAC register)	N/A

Animals and other research organisms

Policy information about studies involving animals; ARRIVE guidelines recommended for reporting animal research, and Sex and Gender in Research

Laboratory animals	The mice used in this study were as follows;<br>NOG mice (In-Vivo Science)<br>NOG/hIL-3/hGM-CSF Tg mice (In-Vivo Science)<br>NOG-W41/hIL-3/hGM-CSF Tg mice (Central Institute for Experimental Animals, Kanagawa, Japan)<br>C57BL/6J (Ly5.1) mice (Sakyo Lab Service)<br>Topors knockout mice (Generated in this study). Topors+/− mice were generated in this study and were backcrossed to a C57BL/6J background for over five generations.<br>All animal experiments were performed in accordance with the institutional guidelines for the use of laboratory animals and approved by the Review Board for Animal Experiments of the University of Tokyo (approval ID, PA18-42).<br>Housing conditions: temperature 22 ± 2°C, humidity 55 ± 5%, light/dark cycle 12hour/12hour (8am-20pm light).
Wild animals	No wild animals were used in this study.
Reporting on sex	NOG mice (In-Vivo Science): 10 weeks old, female<br>NOG/hIL-3/hGM-CSF Tg mice (In-Vivo Science): 10 weeks old, male<br>NOG-W41/hIL-3/hGM-CSF Tg mice (Central Institute for Experimental Animals, Kanagawa, Japan): 14 weeks old, female<br>NOG/hIL-3/hGM-CSF Tg mice (In-Vivo Science): 10 weeks old, female<br>C57BL/6J (Ly5.1) mice (Sakyo Lab Service): 10 weeks old, female<br>Topors knockout mice (Generated in this study) : 2–4 months old, female (male only for the transformation experiment of GMPs by MLL-AF9)
Field-collected samples	No Field-collected samples were used in this study.
Ethics oversight	All experiments using mice were performed in accordance with our institutional guidelines for the use of laboratory animals and approved by the Review Board for Animal Experiments of IMSUT (approval ID: PS18–02).

Note that full information on the approval of the study protocol must also be provided in the manuscript.

Clinical data

Policy information about clinical studies

All manuscripts should comply with the ICMJEguidelines for publication of clinical research and a completedCONSORT checklist must be included with all submissions.

Clinical trial registration	N.A.
Study protocol	N.A.
Data collection	N.A.
Outcomes	N.A.

Flow Cytometry

Plots

Confirm that:

- [x] The axis labels state the marker and fluorochrome used (e.g. CD4-FITC).
- [x] The axis scales are clearly visible. Include numbers along axes only for bottom left plot of group (a 'group' is an analysis of identical markers).
- [x] All plots are contour plots with outliers or pseudocolor plots.
- [x] A numerical value for number of cells or percentage (with statistics) is provided.

Methodology

Sample preparation	Cell lines were cultured as described in the method section. Mouse peripheral blood (PB) cells were collected from the orbital venous plexus using capillaries, and erythrocytes were lysed using erythrocyte lysis buffer (150 mM NH4Cl) before analysis. Bone marrow (BM) cells were extracted from the femur, tibia, and pelvis of euthanized mice. After erythrocyte lysis, BM cells were filtered through a 45 μm filter and centrifuged on a Histopaque-1119 (Sigma) to separate mononuclear cells.
Instrument	FACS Aria III (BD), FACS Celesta (BD)
Software	FlowJo v10
Cell population abundance	Cell sorting efficiency was confirmed by flow cytometric analysis of post-sorted cells.
Gating strategy	FSC/SSC were used for mononuclear and single cell gating. Live cells were gated by PI or DAPI negative.<br>Lineage cells in were defined as Mac-1- and/or Gr-1-positive for the myeloid lineage, CD19-positive for the B cell lineage, or CD3-positive for the T cell lineage. Hematopoietic stem and progenitor cells in BM were defined as lineage-negative, Sca-1-positive, c-Kit-positive for LSK, lineage-negative, Sca-1-negative, c-Kit-positive for myeloid progenitors, and among myeloid progenitors, CD34-positive and FcγRII/III-negative for common myeloid progenitors (CMPs), CD34-negative, FcγRII/III-negative for megakaryocyte-erythrocyte progenitors (MEPs), and CD34-positive, FcγRII/III-positive for granulocyte-macrophage progenitors (GMPs).

☒ Tick this box to confirm that a figure exemplifying the gating strategy is provided in the Supplementary Information.
