# Supplementary material for: Inhibition of TOPORS ubiquitin ligase augments the efficacy of DNA hypomethylating agents through DNMT1 stabilization
Source: Nat Commun. 2024 Aug 28;15:7359. doi: 10.1038/s41467-024-50498-4 (PMC11358161; doi:10.1038/s41467-024-50498-4)

Figure 2d

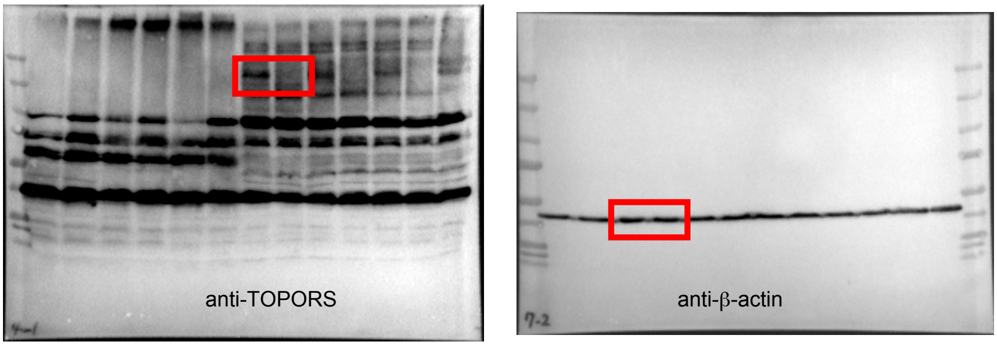

Figure 3b

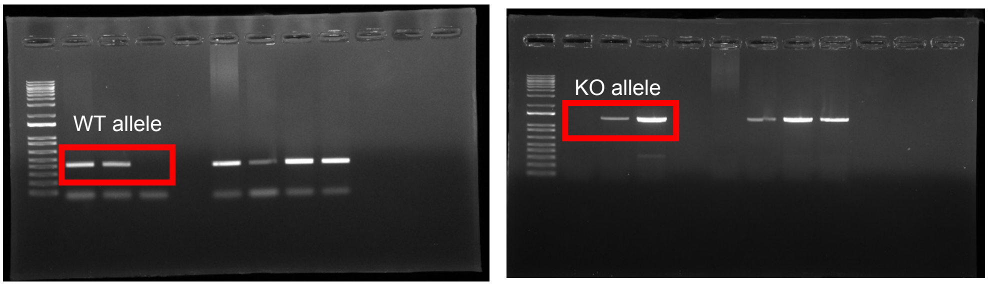

Figure 5e

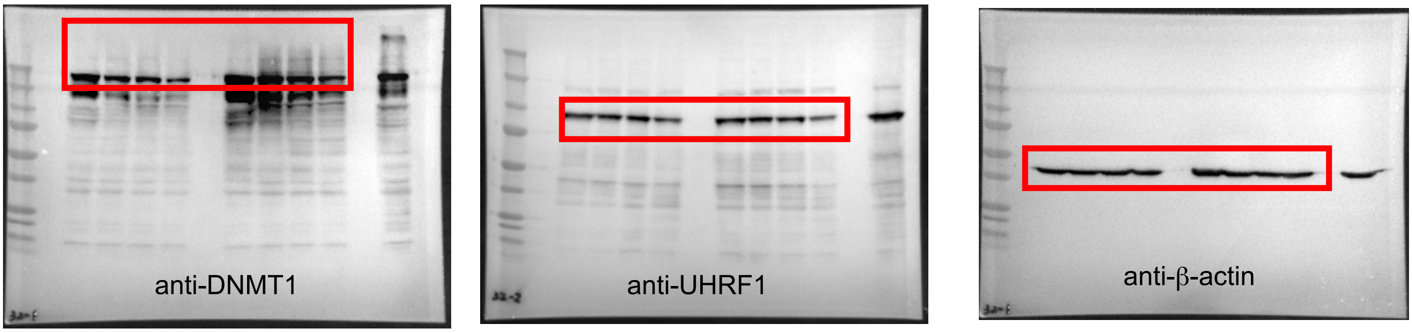

Figure 5f

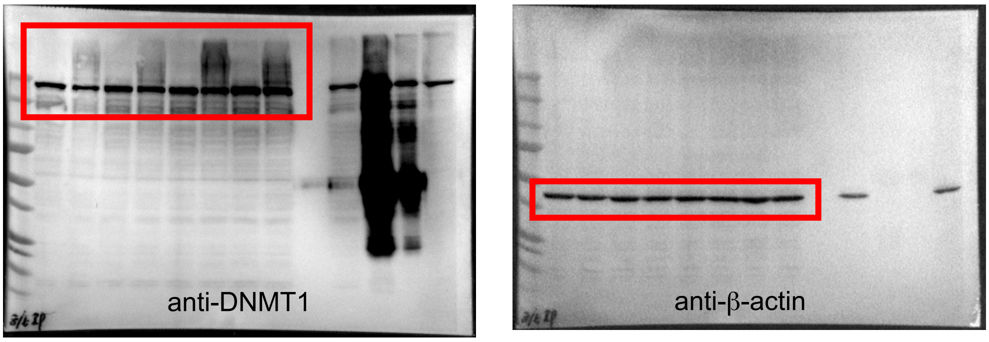

Figure 5g

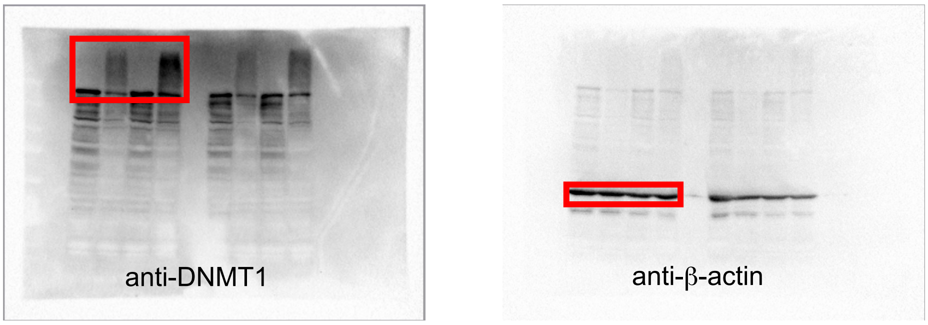

Figure 5h

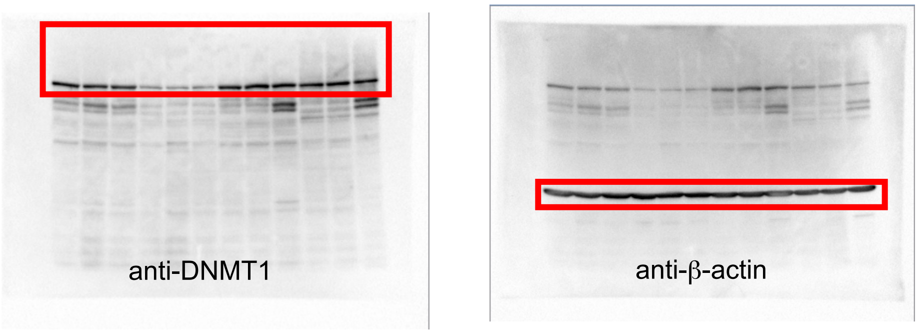

Figure 6d

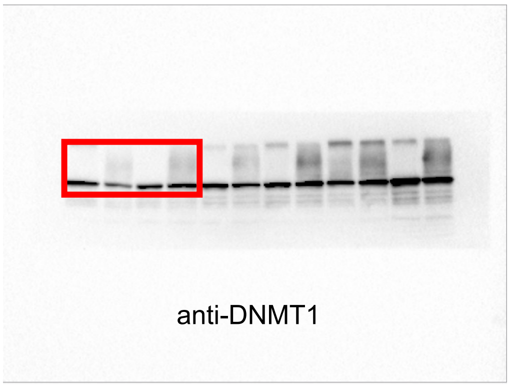

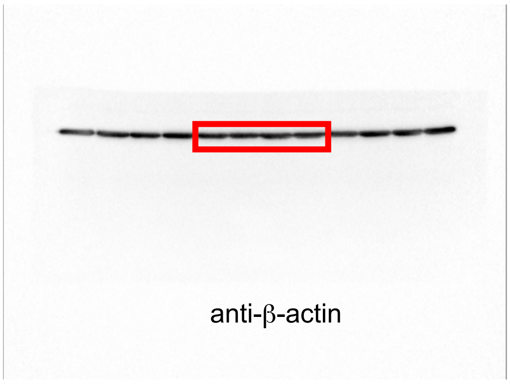

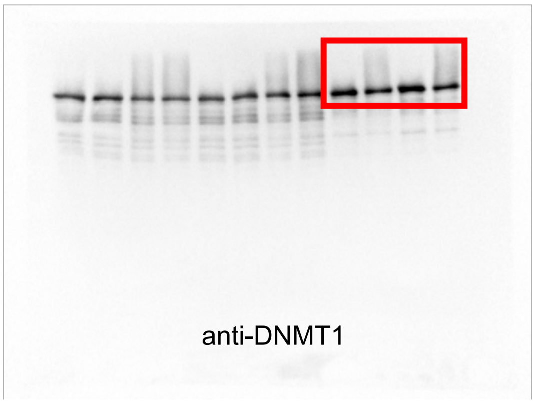

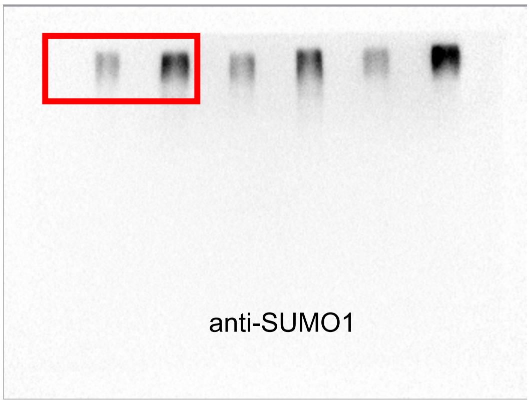

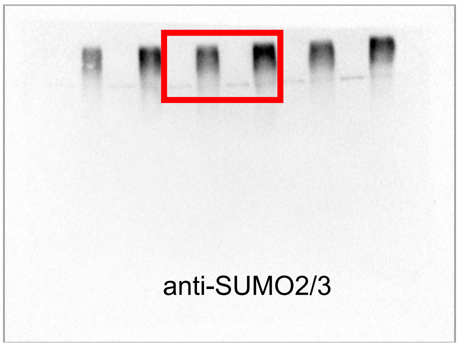

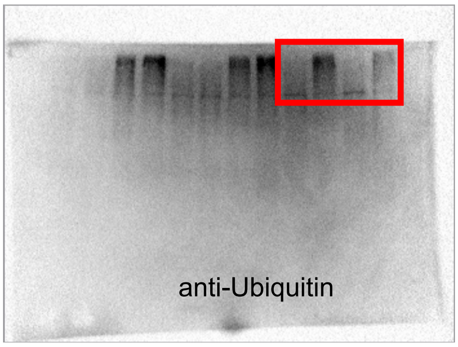

Figure 7a

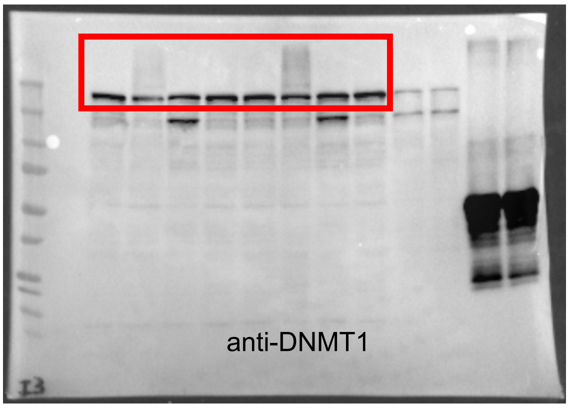

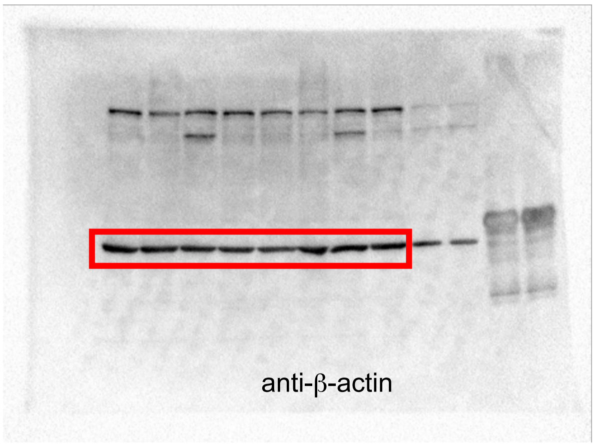

Extended Data Figure 5c

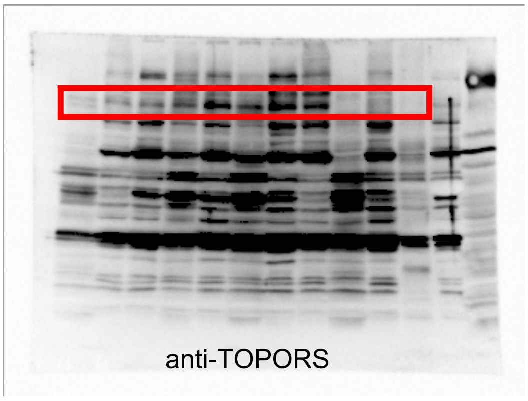

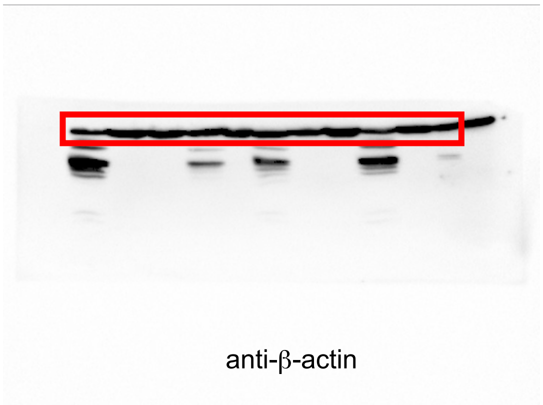

Extended Data Figure 6a

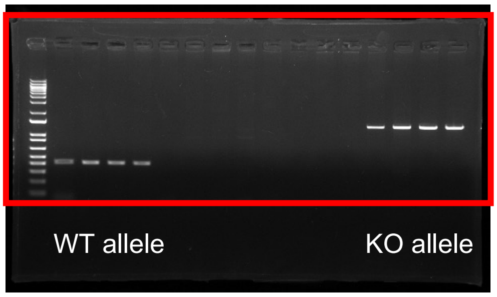

Extended Data Figure 6b

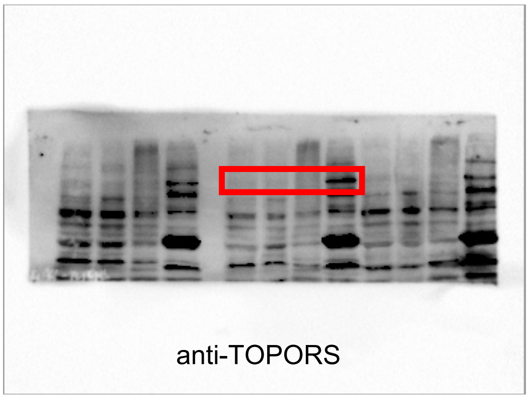

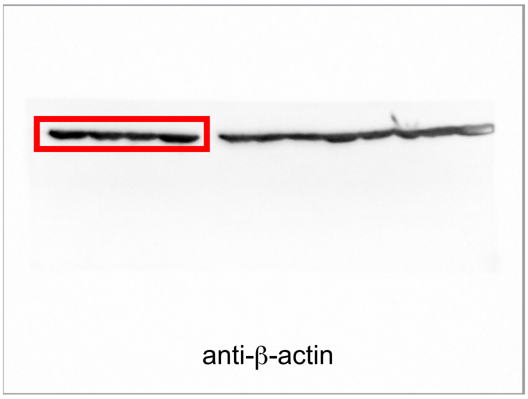

Extended Data Figure 8c

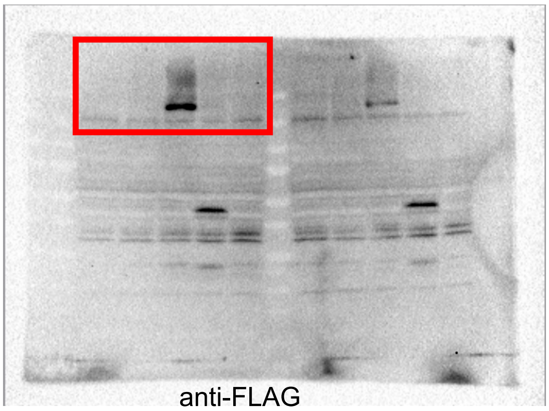

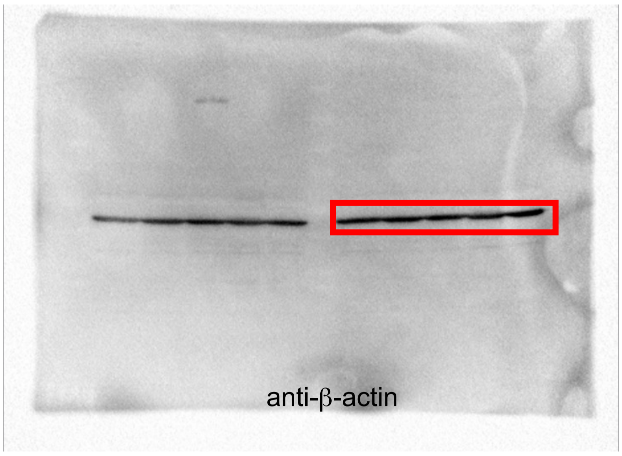

Extended Data Figure 8d

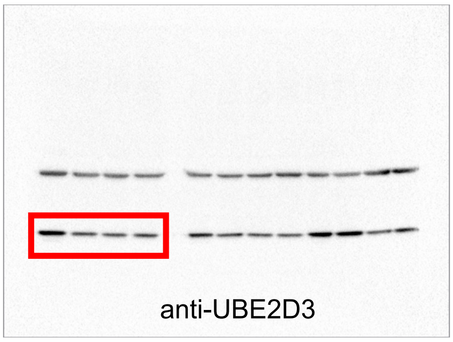

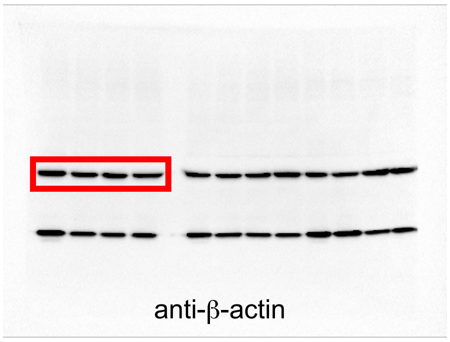

Extended Data Figure 9b

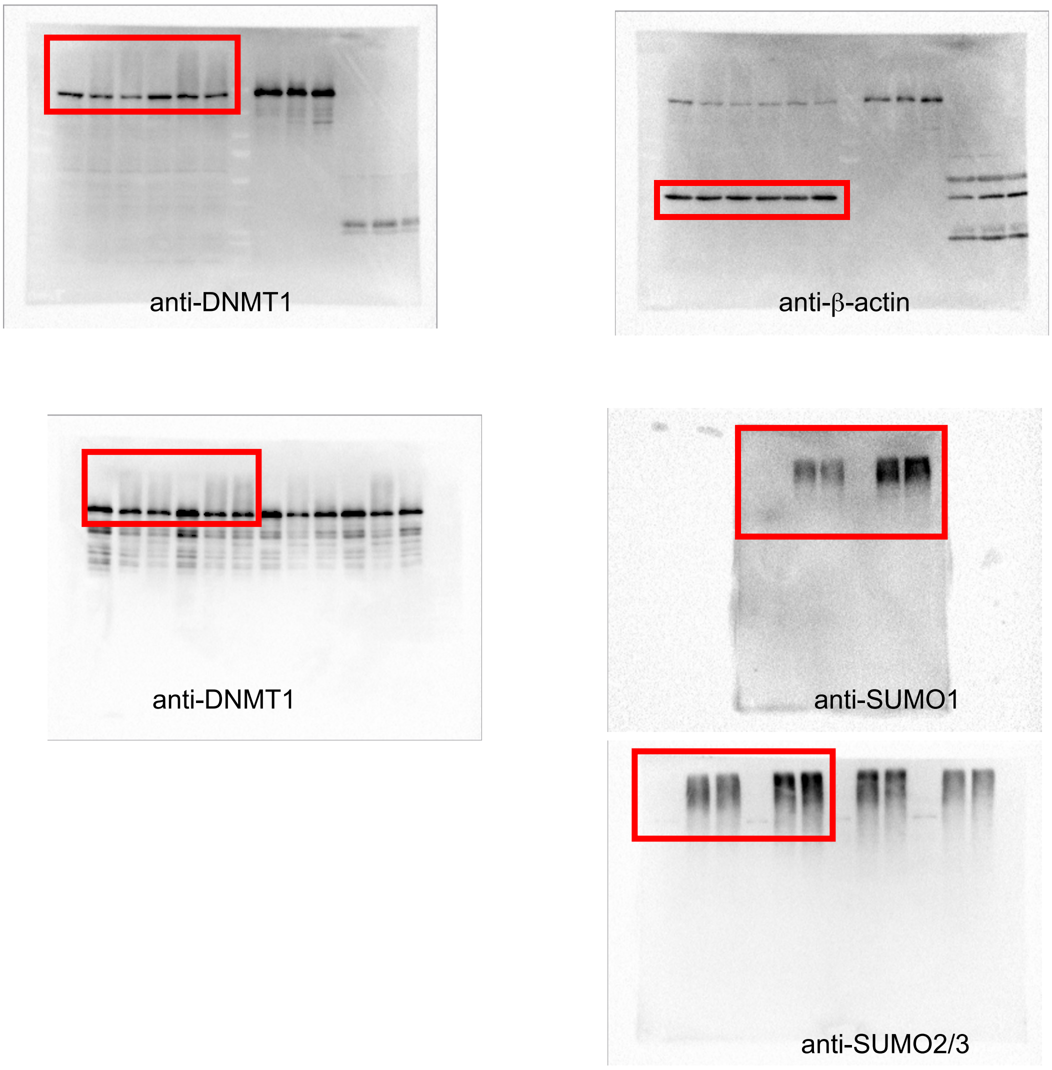

Extended Data Figure 9c

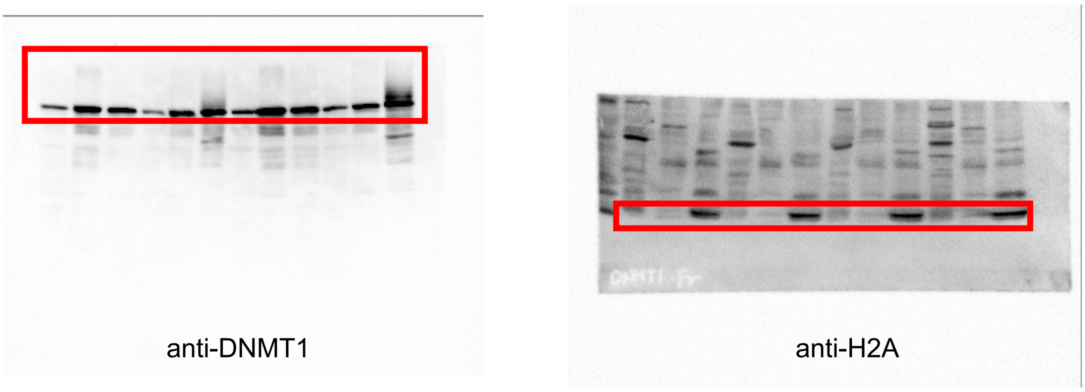

Extended Data Figure 12a

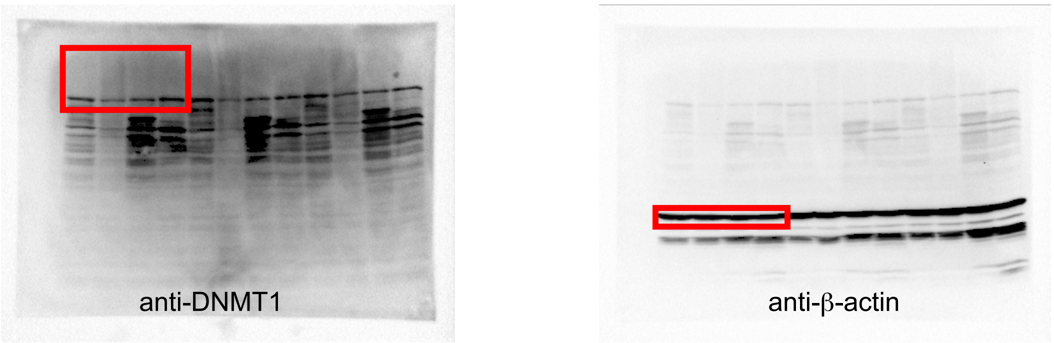

Supplement: Supplementary file 13 — Source data [file 41467_2024_50498_MOESM13_ESM.zip › Source Data/Uncropped images of gels and blots.pdf]
